# Protective Effects of Lipoxin A4 in Testis Injury following Testicular Torsion and Detorsion in Rats

**DOI:** 10.1155/2014/898056

**Published:** 2014-05-08

**Authors:** Xian-Long Zhou, Qi-Sheng Yang, Shao-Zhou Ni, Xiao-Peng Tu, Yan Zhao, Bing Xu, Zheng-Qi Pan, Jun Shen

**Affiliations:** Emergency Center, Zhongnan Hospital of Wuhan University, 169 Donghu Road, Wuchang, Wuhan, Hubei 430071, China

## Abstract

*Purpose*. To investigate the protective effects of lipoxin A4 (LXA4) in rat testis injury following testicular torsion/detorsion. *Methods*. A rat testicular torsion model has been established as described. Rats were randomly divided into 6 groups: sham group, torsion group, torsion/detorsion (T/D) group, and T/D plus LXA4-pretreated groups (3 subgroups). Rats in LXA4-pretreated groups received LXA4 injection (0.1, 1.0, and 10 **μ**g/kg body weight in LXA4-pretreated subgroups 1–3, resp.) at a single dose 1 h before detorsion. Biochemical analysis, apoptosis assessment, and morphologic evaluation were carried out after orchiectomies. *Results*. GPx and SOD levels significantly increased and MDA levels significantly reduced in LXA4-pretreated groups compared to T/D group. LXA4 also reverted IL-2 and TNF-**α** to basal levels and improved the expression of IL-4 and IL-10 in LXA4-pretreated groups. Moreover, the expression of NF-**κ**B was downregulated in LXA4-pretreated groups. LXA4 treatment also showed an improved testicular morphology and decreased apoptosis in testes. *Conclusion*. Lipoxin A4 protects rats against testes injury after torsion/detorsion via modulation of cytokines, oxidative stress, and NF-**κ**B activity.

## 1. Introduction

Testicular torsion occurs at a frequency of 1 in 4000 in men who are younger than 25 years old [1]. It is a common urologic emergency that leads to testicular necrosis and results in decreased fertility [2]. The primary pathophysiologic event in testicular torsion is ischemia followed by reperfusion; thus, testicular torsion/detorsion is considered as an ischemia/reperfusion injury (IRI) to the testis [3]. Numerous drugs have been applied to prevent testis against IRI following testicular torsion in animal models [4–7]. Those previous studies suggested that treatment against IRI following testicular torsion/detorsion may result in decreased oxidative stress, reduced inflammation, and improved testicular morphology to testis.

Lipoxins are lipoxygenase derived lipid mediators with both anti-inflammatory and proresolution properties that have been demonstrated in vivo and in vitro [8]. Lipoxin A4 (LXA4) is the major physiological lipoxin during inflammation in mammalian systems [9]. The protective effects of lipoxins against IRI have been confirmed in many organs including brain, heart, kidney, and stomach [10–13]. However, to our knowledge, the effect of LXA4 on IRI following testicular torsion/detorsion has not yet been reported. In the present study, a rat model was used to investigate the roles of LXA4 in testicular torsion/detorsion.

## 2. Materials and Methods

### 2.1. Animals and Reagents

All experiments were conducted in accordance with the guidelines of Animal Use and Care Committee of Wuhan University. 60~90-day-old specific pathogen-free (SPF) Sprague Dawley (SD) rats weighing 180~200 g were obtained from the Center for Animal Experiment/Animal Biosafety Level III laboratory (ABSL-III lab) of Wuhan University in China. Rats were housed individually in cages on a 12 h dark: 12 h light cycle at 23 ± 2°C under standard environmental conditions and had free access to pellet diet and tap water.

### 2.2. Animal Model and Study Design

Rats were randomly divided into 6 groups: (1) sham group (*n* = 10): rats received sham operations with no additional interventions; (2) torsion group (*n* = 10); (3) torsion/detorsion (T/D) group (*n* = 10) and (4) T/D + LXA4-pretreated groups (*n* = 10 in each subgroup, 3 subgroups in total). The torsion and detorsion protocols lasted for 6 hours. Bilateral orchiectomies were performed at the end of protocol. In detail, bilateral orchiectomies were performed 6 h after sham operation in the sham group; rats in the torsion group received bilateral orchiectomies after 6 h torsion without detorsion; rats in the T/D group and T/D + LXA4 groups received a 2 h torsion followed by a 4 h detorsion. Torsion, detorsion, and sham operations were performed on the left testis through a midscrotal vertical incision as previously described [14]. LXA4 ([5S-, 6R-, 15S-trihydroxy-7E, 9E, 11Z, 13E-eicosatetraenoic acid]; Cayman Chemical, Ann Arbor, USA) was intraperitoneally injected 1 h before detorsion in the T/D + LXA4 groups as a single dose (0.1, 1.0, and 10 *μ*g/kg body weight in subgroup 1–3, resp.). Rats were anesthetized with intraperitoneal injection of pentobarbital sodium (50 mg/kg body weight; Amresco, Cleveland, USA). Tissue samples were collected for biochemical analysis and morphological evaluation at the end of study protocol. Half of each fresh testis was washed with ice-cold phosphate-buffered saline (PBS) (pH = 7.4) and then kept at −70°C for measurement of cytokines levels, MDA level, tissue SOD, and GPx activity. The remaning half of testis was divided into two parts and fixed in 10% formalin or 2.5% glutaraldehyde for light microscopy and electron microscopy, respectively.

### 2.3. Expression of Cytokines in Testes

Cytokines IL4, IL-10, IL-2, and TNF-*α* were detected using commercial ELISA kits according to the manufacturers' protocols (Beyotime Institute of Biotechnology, Jiangsu, China). Each well of the plate was coated with 100 *μ*L of capture antibody and incubated overnight at 4°C. Plates were blocked with assay diluent for 1 h after washes. Testicular tissue homogenate (100 *μ*L) in PBS supplemented with protease inhibitors was added to each well of the plate before incubation. Working detector (100 *μ*L) was loaded into each well, and the plate was incubated for 1 h before the addition of substrate solution. The reaction was stopped by adding 50 *μ*L stop solution. Calculation of the concentrations was performed in a log-log linear regression.

### 2.4. Expressions of Malondialdehyde and Activities of Superoxide Dismutase and Glutathione Peroxidase in Testes

Fresh testicular tissue was placed into a 1.5 mL centrifuge tube. Add 250 *μ*L of RIPA buffer with protease inhibitors. Homogenate was then centrifuged at 11000 ×g for 10 min at 4°C. The supernatant was used for the determination of malondialdehyde (MDA) using a commercial kit (Cayman, Ann Arbor, MI) and the detection of testicular superoxide dismutase (SOD) and glutathione peroxidase (GPx) activities. Testicular SOD activity was measured with an SOD-525TM reagent kit (OXIS International, Foster, CA); the final result was expressed as U/mg protein; tissue GPx activity was measured as described [15]. GPx catalyzes the oxidation of glutathione (GSH) by H_2_O_2_ in a reaction coupled with the conversion of nicotinamide adenine dinucleotide phosphate (NADPH) (reduced form) to NADPH+ (oxidized form), and the change in absorbance at 340 nm is used for detecting GPx activity.

### 2.5. Western-Blot Assay for NF-*κ*B p65

The fresh testicular tissue has been collected and then was homogenized in ice-cold tissue protein extraction reagent, containing 1% protease and 1% phosphatase inhibitors. After centrifugation at 10,000 g for 5 min at 4°C, the supernatants were collected. Protein concentrations in supernatants were determined by a bicinchoninic acid protein assay kit (Boster, Wuhan, China). Western blot assay was carried out in duplicates as described [16]. Anti-NF-kappa B p65 antibody was purchased from Abcam (MA, USA). Protein bands were normalized with GAPDH, and all blots were quantified with Software Quantity One (Bio Rad).

### 2.6. Assessment of Apoptosis in Testes

Apoptotic activity on paraffin sections of the testis was analyzed by a TUNEL method with a commercial kit (Boster, Wuhan, China). The number of TUNEL-positive nuclei per tubule was counted. Apoptotic cells were identified by a brown stain over the nuclei. Approximately, 200 cells were counted per field; five fields were examined per slide. Apoptotic index (AI) was calculated as follows: AI = (number of apoptotic cells/the total number of counted cells) × 100%. Caspase-3 is activated in the apoptotic cell both by extrinsic (death ligand) and intrinsic (mitochondrial) pathways. We also measured the caspase-3 protease activity using a caspase-3 colorimetric assay kit according to the manufacturer's instructions (Boster, Wuhan, China). Briefly, the homogenates of testicular tissues were centrifuged for 1 min at 10,000 g, and 100 *μ*g of protein was incubated with reaction buffer and Asp-Glu-Val-Asp-p-nitroanilide for 90 min at 37°C. Absorbance was measured at 405 nm as caspase-3 activity.

### 2.7. Morphological Evaluation

Testis tissues were collected for morphological evaluation. The specimens were fixed in 10% formalin, embedded in paraffin, cut into sections 4 microns in thickness, and stained with hematoxylin and eosin (H&E). A pathologist blindly evaluated the testicular tissues in a random order under light microscope. Testicular injury and spermatogenesis were graded with Johnsen score [17]. All tubular sections in each observed area of testicular tissue are evaluated systematically and each is given a score from 1 to 10. Complete spermatogenesis with many spermatozoa present is evaluated as score 10. Additionally, electron microscopy was also carried out. Briefly, testicular tissue was fixed in 2.5% glutaraldehyde at 4°C for 24 h then washed in phosphate-buffered saline, embedded in epoxy resin, and immersed in Epon812. Sections collected with a LKB-V microtome (BROMMA, Sweden) were stained with uranium acetate and folic acid lead and captured with a transmission electron microscope (H-600, Hitachi, Japan).

### 2.8. Statistical Analysis

Data are expressed as mean ± SD. The data was processed by the statistical analysis software SPSS version 16.0 (SPSS Inc., Chicago, IL). Comparison of several means was performed using one-way and repeated measure two-way analysis of variance followed by the Tukey-Kramer test to identify significant difference between groups. All *P* values were two-tailed and a *P* value of less than 0.05 was considered significant.

## 3. Results

### 3.1. Changes of Cytokines, MDA, SOD, and GPx in Testis

**Figure 1:**
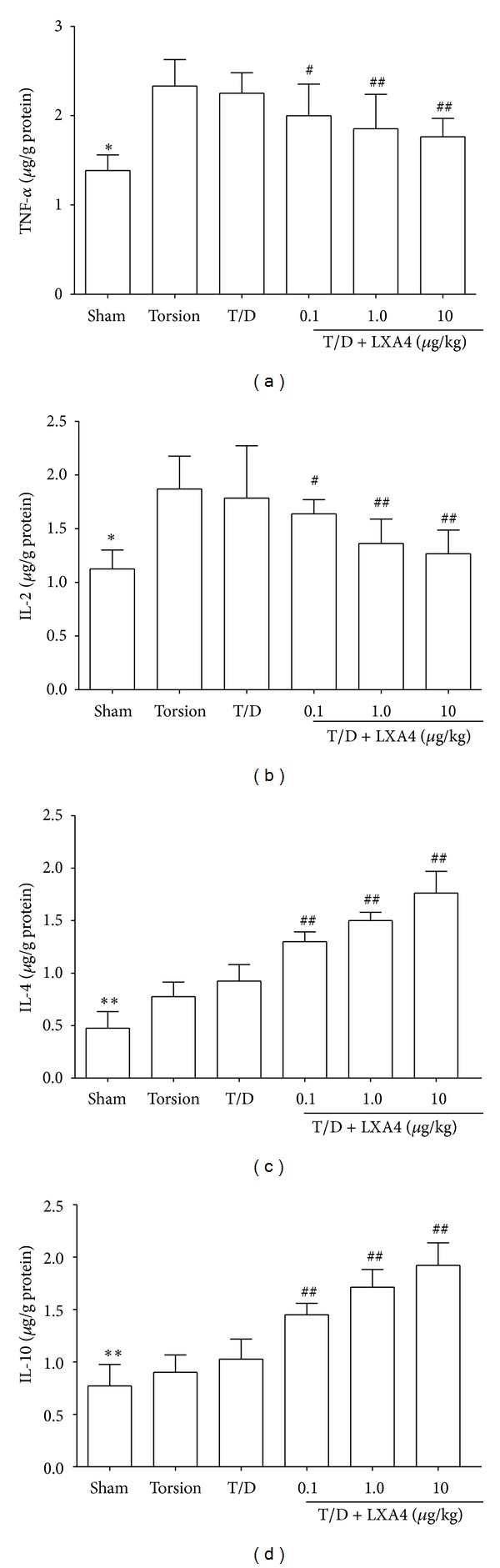
Changes of cytokines in testicular tissue. LXA4 treatment inhibits the increase of proinflammatory cytokines (IL-2 and TNF-*α*) and promotes the expression of anti-inflammatory cytokines (IL-4 and IL-10) in testes after testicular torsion. Compared with other groups, *P < 0.05 and **P < 0.01. Compared with torsion or torsion/detorsion (T/D) group, ^#^ *P* < 0.05 and ^##^ *P* < 0.01.

**Figure 2:**
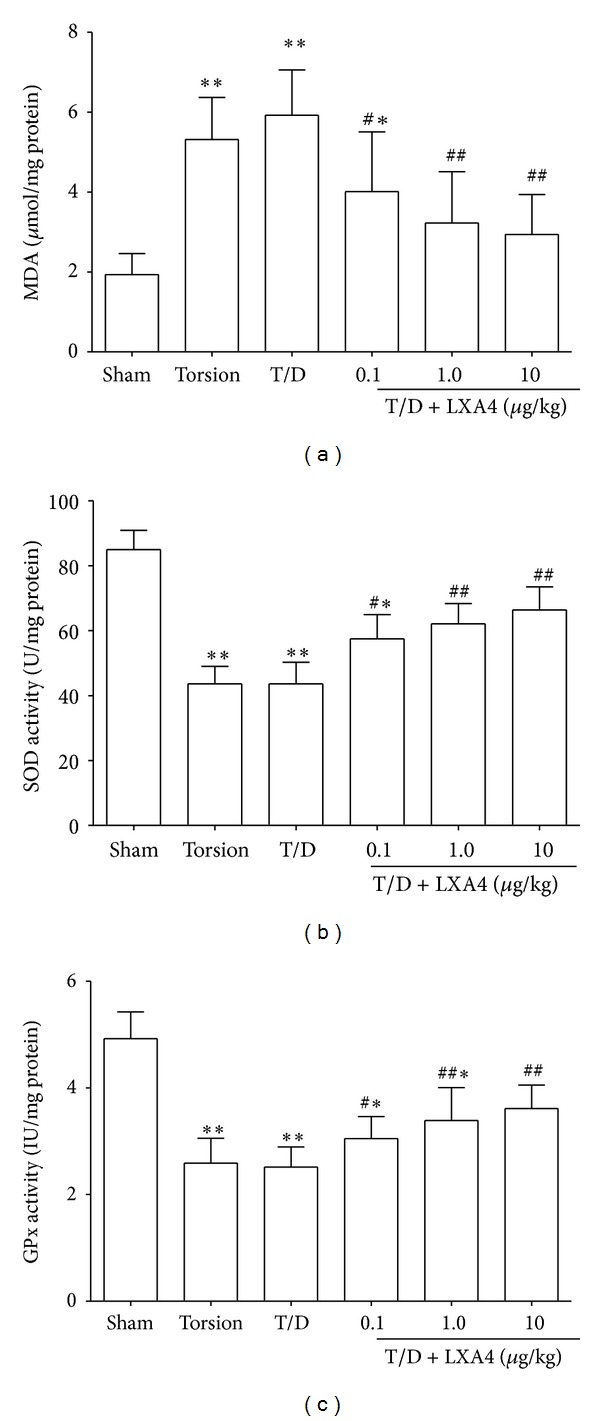
Changes of MDA, SOD, and GPx in testicular tissue. The levels of MDA were increased in testes after testicular torsion, while the levels of SOD and GPx were decreased. The treatment with LXA4 reverted those parameters to basal levels. Compared with Sham groups, *P < 0.05 and **P < 0.01. Compared with torsion or torsion/detorsion (T/D) group, ^#^ *P* < 0.05 and ^##^ *P* < 0.01.

Changes of cytokines, MDA, SOD, and GPx were summarized in Figures 1 and 2. Compared to the sham group, the levels of proinflammatory cytokines (IL-2 and TNF-*α*) and MDA were significantly increased in testes after testicular torsion, while the levels of SOD and GPx were decreased (resp., *P* < 0.05). The treatment with LXA4 reverted those parameters to basal levels. Compared to the torsion and torsion/detorsion group, the MDA and IL-2 and TNF-*α* levels were significantly lower in the LXA4-pretreated groups (resp., *P* < 0.05). In addition, the anti-inflammatory cytokines (IL-4 and IL-10), SOD, and GPx levels were significantly increased in the LXA4-pretreated groups than those in the torsion and torsion/detorsion groups (resp., *P* < 0.05).

### 3.2. NF-*κ*B p65 Expression in Testis

**Figure 3:**
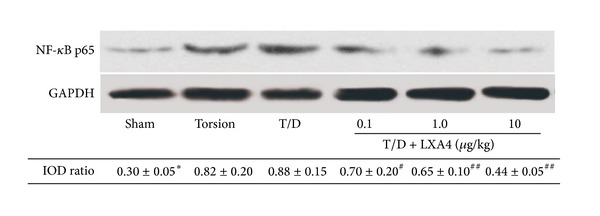
Western-blot for NF-*κ*B p65 expression in testicular tissue. NF-*κ*B p65 expression was increased after testicular torsion in both torsion and torsion/detorsion (T/D) groups. However, the NF-*κ*B p65 expression has been inhibited by LXA4 treatments. Compared with other groups, *P < 0.05. Compared with T/D group, ^#^ *P* < 0.05 and ^##^ *P* < 0.01.

Western-blot assay showed that the NF-*κ*B p65 expression level was increased after testicular torsion. However, the NF-*κ*B p65 expression has been inhibited by LXA4 treatment. As seen in Figure 3, the NF-*κ*B p65 expressions in LXA4-treated groups were significantly lower than those in the torsion and torsion/detorsion groups (resp., *P* < 0.05).

### 3.3. Analysis of Apoptosis in Testis

**Figure 4:**
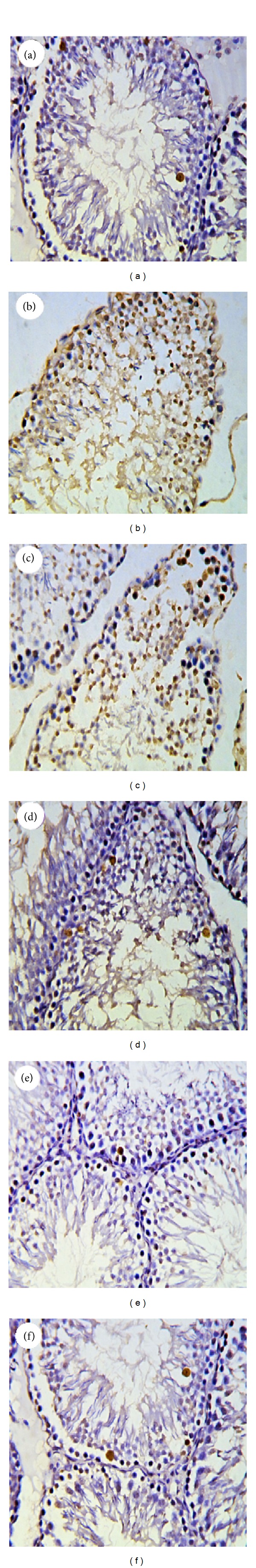
TUNEL staining of testicular tissue (×200). (a) Sham group: only a few TUNEL-positive cells were observed. (b) Torsion group and (c) torsion/detorsion (T/D) group: TUNEL-positive cells were mainly observed in germ cells of testis. (d), (e), and (f): T/D + LXA4 subgroup 1–3, respectively. Apoptotic cells were rarely observed in the seminiferous epithelium.

The analysis of apoptosis was performed by TUNEL method and caspase-3 activity. TUNEL staining sections were shown in Figure 4. The percentage of TUNEL positive apoptotic cells was denoted as AI. The AI values of LXA4-pretreated groups were significantly lower than those in the torsion and torsion/detorsion groups (resp., *P* < 0.05, Table 1). In addition, compared to torsion and torsion/detorsion groups, the caspase-3 activity in testes was significantly decreased in the LXA4-pretreated groups (resp., *P* < 0.05, Table 1).

### 3.4. Morphological Evaluation of Testis

**Figure 5:**
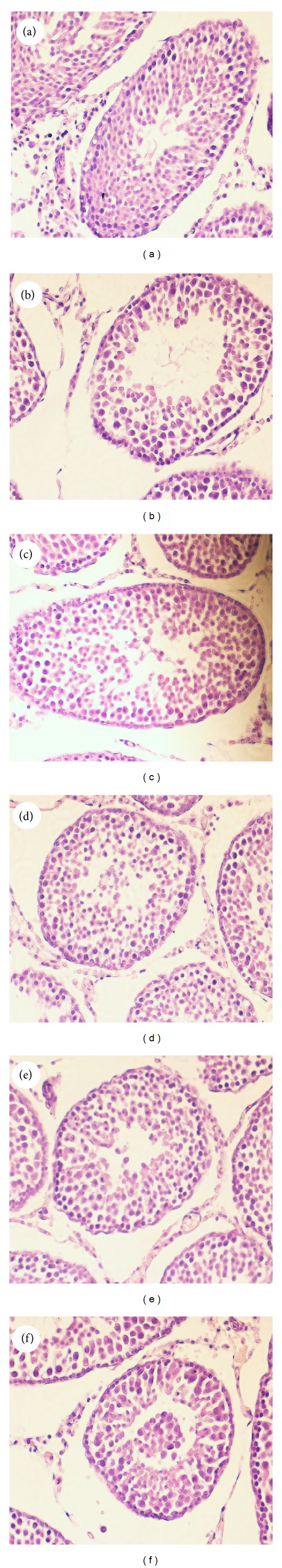
Light microscope observations of H&E stained sections (×200). (a) Sham group: normal testicular architecture was seen. (b) Torsion group and (c) torsion/detorsion (T/D) group: severe damage to testis was noted. (d), (e), and (f): T/D + LXA4 subgroup 1–3, respectively.

**Figure 6:**
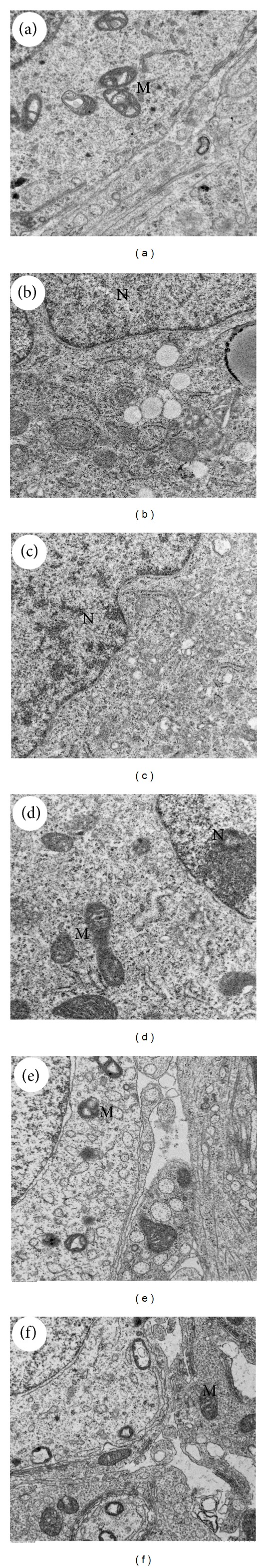
Electron microscopy of testicular tissue in different groups (×2000), N: nuclei, and M: mitochondria. (a) Sham group: normal testicular structure was seen in sham group. (b) Torsion group and (c) torsion/detorsion (T/D) group: swollen mitochondria with degenerated cristae and enlarged intercellular spaces were observed under electron microscopy. (d), (e), and (f): T/D + LXA4 subgroup 1–3, respectively: LXA4 pretreatment was effective in preventing mitochondria degeneration and dilation of intracellular spaces.

The findings of the light microscopy for each group are shown in Figure 5. The presence of normal testicular structure and uniform seminiferous tubular morphology was seen in the sham group. In the torsion and T/D groups, there were significant reductions in the seminiferous tubular diameter and severe distortion of tubules. Administration of LXA4 preserved the intact seminiferous tubular morphology in testes after torsion/detorsion. Furthermore, the histologic scores were significantly higher in the LXA4-treated groups compared with the torsion and torsion/detorsion groups (resp., *P* < 0.05, Table 1). Swollen mitochondria with degenerated cristae and enlarged intercellular spaces were observed under electron microscopy in both torsion and T/D groups. However, LXA4 pretreatment was effective in preventing mitochondria degeneration and dilation of intracellular spaces (Figure 6).

## 4. Discussion

In this study, we used a rat testicular torsion model to investigate the protective effects of lipoxin A4 on testicular ischemia/reperfusion injury. Various parameters such as MDA, SOD, GPx, proinflammatory cytokines, anti-inflammatory cytokines, and NF-*κ*B have been detected. Morphologic evaluation has been also carried out after orchiectomies. Our results demonstrated that lipoxin A4 significantly reduced the inflammatory reactions, oxidative stress, and histologic damage in testes after testicular torsion.

The ischemia of the testes followed by reperfusion is the primary pathophysiological event in testicular torsion (TT) [18]. Thus, TT can be generally considered as an ischemia/reperfusion injury (IRI) to testis. Numerous studies focused on the treatment against IRI following testicular torsion and detorsion have brought beneficial effects in animal model [14, 19, 20]. The protective effects of lipoxins against IRI have been confirmed in many organs including brain, heart, kidney, and stomach [10–13]. In this study, LXA4 also showed its protective effects in the testicular IRI due to its ability of modulation of oxidative stress and inflammation.

IRI to testis is associated with the overgeneration of reactive oxygen species (ROS) and mammalian testes are highly susceptible to oxidative stress [19, 20]. MDA has been widely used as an indicator of oxidative stress in many physiopathological events including IRI [21]. Many studies proved that MDA level in testis tissue increase after testicular torsion [22]. Besides, the ability of antioxidants including SOD and GPx to prevent testis against IRI following testicular torsion has been investigated [23, 24]. In this study, treatment with LXA4 attenuated the oxidative stress in damaged testes via reducing the expression of MDA and elevating the expression of SOD and GPx. Those results showed that LXA4 might exert an antioxidative effect in testicular detorsion.

Inflammation has been established to contribute substantially to the pathogenesis of IRI. The proinflammatory cytokines such as tumor necrosis factor-*α* (TNF-*α*) and interleukin-1*β* (IL-1*β*) increase in IRI following testicular torsion [16]. LXA4 has been considered as a “break signal” of inflammation, and the role for LXA4 as anti-inflammatory molecules is well defined [25]. In this study, LXA4-treated rats showed lower proinflammatory cytokine levels compared to the torsion/detorsion group. Our results also suggested that the expression of NF-*κ*B in damaged testes has been downregulated by the LXA4 pretreatment. Nuclear factor *κ*B (NF-*κ*B) is a primary regulator of gene expression for a large number of cytokines and is activated during IRI of testis [26], and LXA4 have been proposed to directly stimulate gene expression of endogenous anti-inflammatory factors by regulating NF-*κ*B activation [11]. It is suggested that the protective effect of LXA4 in testicular torsion followed IRI was partially due to its anti-inflammatory properties.

Experimental studies have shown that bilateral testicular damage and reduced fertility may result from unilateral torsion [27]. The proposed mechanism of this injury is probably due to the immune response after testicular torsion. In addition, animal experiments suggested that humoral and cellular immune mediated testicular cell damage is an important pathologic alternation in testicular torsion [28]. A few recent studies have demonstrated that lipoxins suppress antigen-presenting cell functions and regulate cytokine-driven immune reactions toward Th2 responses [29–31]. The Th1 subset mainly secretes IL-2, IFN-*γ*, and TNF-*α*. In contrast, Th2 cells mainly produce IL-4 and IL-10. In this study, we observed that the anti-inflammatory cytokines IL-4 and IL-10 were significantly increased in the LXA4-pretreated groups, while IL-2 and TNF-*α* were decreased. These findings suggested that LXA4 attenuates the IRI following testicular torsion and is also associated with its ability of regulating Th1/Th2 balance.

## 5. Conclusion

Our results suggest that LXA4 may exert an anti-inflammatory effect and reduce the histological damage in rat testicular torsion/detorsion due to its ability to regulate the production of cytokines and the NF-*κ*B activity and to cause MDA, SOD, and GPx to revert to control levels. Thus, LXA4 may have a protective effect against IRI injury following testicular torsion/detorsion via modulation of cytokines, oxidative stress, and NF-*κ*B activity.

## Figures and Tables

**Table 1:** Apoptotic index, Johnsen scores, and caspase-3 activities in different groups (mean ± SD).

	Apoptotic index (%)	Johnsen scores	Caspase-3 activity (absorbance 405/100 *μ*g protein)
Sham	8.50 ± 4.10**	7.50 ± 0.80*	0.10 ± 0.00**
Torsion	32.40 ± 7.10	5.22 ± 1.21	0.62 ± 0.10
T/D	30.52 ± 10.20	5.50 ± 1.00	0.55 ± 0.05
T/D + LXA4 (0.1 *μ*g/kg)	24.60 ± 7.70^#^	5.80 ± 1.00^#^	0.42 ± 0.12^##^
T/D + LXA4 (1.0 *μ*g/kg)	18.50 ± 8.12^##^	6.22 ± 1.40^##^	0.35 ± 0.08^##^
T/D + LXA4 (10 *μ*g/kg)	14.30 ± 6.75^##^	7.15 ± 1.50^##^	0.22 ± 0.05^##^

T/D: torsion/detorsion; LXA4: lipoxin A4. Testicular injury and spermatogenesis were graded with Johnsen score. All tubular sections in each observed area of testicular tissue are evaluated systematically and each is given a score from 1 to 10. Complete spermatogenesis with many spermatozoa present is evaluated as score 10. Compared with other groups, *P < 0.05 and **P < 0.01. Compared with torsion or torsion/detorsion (T/D) group, ^#^ *P* < 0.05 and ^##^ *P* < 0.01.

## References

[1] Williamson RC (1976). Torsion of the testis and allied conditions. *British Journal of Surgery*.

[2] Bozlu M, Acar D, Cayan S, Aktas S, Tunckiran A (2009). Protective effect of trapidil on long-term histologic damage in a rat model of testicular ischemia-reperfusion injury. *World Journal of Urology*.

[3] Dokmeci D (2006). Testicular torsion, oxidative stress and the role of antioxidant therapy. *Folia medica*.

[4] Dokmeci D, Kanter M, Inan M (2007). Protective effects of ibuprofen on testicular torsion/detorsion-induced ischemia/reperfusion injury in rats. *Archives of Toxicology*.

[5] Romeo C, Antonuccio P, Esposito M (2004). Raxofelast, a hydrophilic vitamin e-like antioxidant, reduces testicular ischemia-reperfusion injury. *Urological Research*.

[6] Akgül T, Ayyildiz A, Nuhoğlu B (2008). Ginkgo biloba (EGb 761) usage attenuates testicular injury induced by testicular ischemia/reperfusion in rats. *International Urology and Nephrology*.

[7] Tsounapi P, Saito M, Dimitriadis F (2011). Protective effect of sivelestat, a neutrophil elastase inhibitor, on ipsilateral and contralateral testes after unilateral testicular ischaemia-reperfusion injury in rats. *BJU International*.

[8] Serhan CN, Hamberg M, Samuelsson B (1984). Lipoxins: novel series of biologically active compounds formed from arachidonic acid in human leukocytes. *Proceedings of the National Academy of Sciences of the United States of America*.

[9] Baker N, O’Meara SJ, Scannell M, Maderna P, Godson C (2009). Lipoxin A4: anti-inflammatory and anti-angiogenic impact on endothelial cells. *The Journal of Immunology*.

[10] Wu Y, Wang Y-P, Guo P (2012). A lipoxin A4 analog ameliorates blood-brain barrier dysfunction and reduces MMP-9 expression in a rat model of focal cerebral ischemia-reperfusion injury. *Journal of Molecular Neuroscience*.

[11] Chen Z, Wu Z, Huang C (2013). Effect of lipoxin A4 on myocardial ischemia reperfusion injury following cardiac arrest in a rabbit model. *Inflammation*.

[12] Kieran NE, Doran PP, Connolly SB (2003). Modification of the transcriptomic response to renal ischemia/reperfusion injury by lipoxin analog. *Kidney International*.

[13] Peskar BM, Ehrlich K, Schuligoi R, Peskar BA (2009). Role of lipoxygenases and the lipoxin A4/annexin 1 receptor in ischemia-reperfusion-induced gastric mucosal damage in rats. *Pharmacology*.

[14] Somuncu S, Cakmak M, Erdogan S, Caglayan O, Akman H, Kaya M (2006). Protective effects of trapidil in ischemia-reperfusion injury due to testicular torsion and detorsion: an experimental study. *International Journal of Urology*.

[15] Paglia DE, Valentine WN (1967). Studies on the quantitative and qualitative characterization of erythrocyte glutathione peroxidase. *The Journal of Laboratory and Clinical Medicine*.

[16] Zhang Y, Lv Y, Liu YJ (2013). Hyperbaric oxygen therapy in rats attenuates ischemia-reperfusion testicular injury through blockade of oxidative stress, suppression of inflammation, and reduction of nitric oxide formation. *Urology*.

[17] Johnsen SG (1970). Testicular biopsy score count—a method for registration of spermatogenesis in human testes: normal values and results in 335 hypogonadal males. *Hormones*.

[18] Turner TT, Brown KJ (1993). Spermatic cord torsion: loss of spermatogenesis despite return of blood flow. *Biology of Reproduction*.

[19] Wei S-M, Yan Z-Z, Zhou J (2007). Beneficial effect of taurine on testicular ischemia-reperfusion injury in rats. *Urology*.

[20] Dokmeci D, Inan M, Basaran UN (2007). Protective effect of L-carnitine on testicular ischaemia-reperfusion injury in rats. *Cell Biochemistry and Function*.

[21] Fukuda K, Asoh S, Ishikawa M, Yamamoto Y, Ohsawa I, Ohta S (2007). Inhalation of hydrogen gas suppresses hepatic injury caused by ischemia/reperfusion through reducing oxidative stress. *Biochemical and Biophysical Research Communications*.

[22] Unsal A, Eroglu M, Avci A (2006). Protective role of natural antioxidant supplementation on testicular tissue after testicular torsion and detorsion. *Scandinavian Journal of Urology and Nephrology*.

[23] Avlan D, Erdouğan K, Cimen B, Düşmez Apa D, Cinel I, Aksöyek S (2005). The protective effect of selenium on ipsilateral and contralateral testes in testicular reperfusion injury. *Pediatric Surgery International*.

[24] Filho DW, Torres MA, Bordin AL, Crezcynski-Pasa TB, Boveris A (2004). Spermatic cord torsion, reactive oxygen and nitrogen species and ischemia-reperfusion injury. *Molecular Aspects of Medicine*.

[25] Serhan CN, Oliw E (2001). Unorthodox routes to prostanoid formation: new twists in cyclooxygenase-initiated pathways. *The Journal of Clinical Investigation*.

[26] Lysiak JJ, Bang HJ, Nguyen QA, Turner TT (2005). Activation of the nuclear factor kappa B pathway following ischemia-reperfusion of the murine testis. *Journal of Andrology*.

[27] Rabinowitz R, Nagler H, Kogan S, Consentino M (1985). Experimental aspects of testicular torsion. *Dialogues in Pediatric Urology*.

[28] Rodriguez MG, Rival C, Theas MS, Lustig L (2006). Immunohistopathology of the contralateral testis of rats undergoing experimental torsion of the spermatic cord. *Asian Journal of Andrology*.

[29] Machado FS, Aliberti J (2009). Lipoxins as an immune-escape mechanism. *Advances in Experimental Medicine and Biology*.

[30] Parkinson JF (2006). Lipoxin and synthetic lipoxin analogs: an overview of anti-inflammatory functions and new concepts in immunomodulation. *Inflammation & Allergy: Drug Targets*.

[31] Liao W, Zeng F, Kang K (2013). Lipoxin A4 attenuates acute rejection via shifting TH1/TH2 cytokine balance in rat liver transplantation. *Transplantation Proceedings*.

